# Despite Buffers, Experimental Forest Clearcuts Impact Amphibian Body Size and Biomass

**DOI:** 10.1371/journal.pone.0143505

**Published:** 2015-11-23

**Authors:** Jessica S. Veysey Powell, Kimberly J. Babbitt

**Affiliations:** Department of Natural Resources and the Environment, University of New Hampshire, Durham, New Hampshire, United States of America; Curtin University, AUSTRALIA

## Abstract

Forest buffers are a primary tool used to protect wetland-dependent wildlife. Though implemented widely, buffer efficacy is untested for most amphibian species. Consequently, it remains unclear whether buffers are sufficient for maintaining amphibian populations and if so, how wide buffers should be. We present evidence from a six-year, landscape-scale experiment testing the impacts of clearcutting, buffer width, and hydroperiod on body size and condition and biomass of breeding adults for two amphibian species at 11 vernal pools in the northeastern United States. We randomly assigned treatments (i.e., reference, 100m buffer, 30m buffer) across pools, clearcut to create buffers, and captured all spotted salamanders and wood frogs. Clearcuts strongly and negatively impacted size, condition, and biomass, but wider buffers mitigated effect magnitude and duration. Among recaptured individuals, for example, 30m-treatment salamanders were predicted to be about 9.5 mm shorter than, while 100m-treatment salamanders did not differ in length from, reference-treatment salamanders. Similarly, among recaptured frogs, mean length in the 30m treatment was predicted to decrease by about 1 mm/year, while in the 100m and reference treatments, length was time-invariant. Some, but not all, metrics recovered with time. For example, female new-captured and recaptured salamanders were predicted, respectively and on average, to weigh 4.5 and 7 g less in the 30m versus reference treatment right after the cut. While recaptured-female mass was predicted to recover by 9.5 years post-cut, new-captured-female mass did not recover. Hydroperiod was an important mediator: in the 100m treatment, cutting predominately affected pools that were stressed hydrologically. Overall, salamanders and female frogs were impacted more than male frogs. Our results highlight the importance of individualized metrics like body size, which can reveal sublethal effects and illuminate mechanisms by which habitat disturbance impacts wildlife populations. Individualized metrics thus provide critical insights that complement species occurrence and abundance-based population assessments.

## Introduction

Globally, forest ecosystems are experiencing intensifying stress as growing human populations demand more developed and agricultural land and larger volumes of forest products [1, 2]. Whether owners develop, harvest, or conserve their forests is a complex decision driven by global economic trends [1, 3]. Over the last two decades, increasing awareness that forests provide critical ecosystem services catalyzed interest in sustainable management programs that allow forest owners to harvest timber while maintaining ecosystem functions and biodiversity [4–6]. Developing sustainable forest-management plans can be difficult even for common species, however, given our sometimes rudimentary understanding of the complex interactions between forest components and ability to predict species’ responses to disturbance [7].

Amphibians can be particularly challenging to accommodate given their complex life cycles and diverse habitat needs [8]. In temperate forests, many amphibian species occupy wetlands during their egg and larval stages, but migrate hundreds of meters into adjacent forest as juveniles and adults (e.g., [9–11]). Forest harvesting can alter both the wetland and upland habitat of these species, with potentially negative consequences for population persistence [8]. In general, timber harvesting, especially clearcuts, is locally associated with reduced abundance and survival of numerous amphibian species across various forest types (e.g., [12–14]). Responding to such scientific evidence, public pressure, economic incentive, and personal ecological ethic, some forest managers in temperate ecosystems have indicated willingness to integrate amphibian habitat needs into forest management plans [15–17].

Forested buffers are a primary tool used to protect amphibians in such plans. Though buffers are implemented widely, their efficacy is untested for most amphibian species. Thus, it remains unclear whether buffers are sufficient for maintaining viable amphibian populations in working forests and if so, how wide buffers should be. Most studies that recommend amphibian buffers are based on observational data from unbuffered landscapes (e.g., [18–20]). After reviewing the movement characteristics of 32 species across such landscapes, Semlitsch and Bodie [9] suggested that a 290-m life zone, centered like a buffer around a wetland, is necessary to protect the core habitat of most wetland-dependent amphibian species. Scientists and conservation planners frequently reference the need for a protective 290-m life zone, but policy-makers are slow to embrace such large constraints on land use [21–23]. Compared to development and intensive agriculture, however, forestry can be a temporary disturbance. Because forests typically regenerate for several decades post-cut, habitat conditions are dynamic and amphibians may be able to persist even if buffers considerably smaller than 290 m are used [14, 21, 24].

Only a handful of studies have intentionally tested the impacts of buffer-mediated forest cutting on amphibians, however, and these have limited inference. Most were restricted to stream-side habitats (e.g., [25, 26]), used narrow buffers (i.e., <35 m; e.g., [27–29]), and were conducted in northwestern North America. For some, forestry impacts were confounded by other management treatments or time of harvest [30, 31]. Some focused solely or partly on terrestrial species [29, 32] or only sampled in or extremely close to streams [33, 34]. SSSuch studies have limited applicability for amphibians that breed in lentic habitats, especially since post-breeding migrations for such species often extend far beyond 35 m.

To strengthen the scientific basis for making decisions about buffer width, we present evidence from a six-year, landscape-scale experiment testing the interactive impacts of clearcutting and buffer width on breeding-adult demography for two amphibian species at natural vernal pools in an industrial forest in the northeastern United States. To our knowledge, this is the first experiment to evaluate buffer efficacy for pool-breeding amphibians. In a previous paper issuing from this experiment [35], we showed that narrow buffers result in reduced recaptures of mature spotted salamanders (*Ambystoma maculatum*) and wood frogs (*Lithobates sylvaticus*) and altered sex ratios for spotted salamanders. Here, we assess how body size and condition and population biomass vary in response to buffer width for breeding adults of both species. Note that competitive and predaceous interactions between larvae of these two species can influence individual body size and condition and population biomass [36–38], but we assumed such influence was comparable across pools (because both species were abundant at all pools) and did not assess interspecies interactions in this study.

Amphibian body size and condition are correlated with and can be proxies for multiple fitness measures including fecundity [39, 40], survival [41, 42], endurance [43, 44], and immunity [45, 46]. Biomass measures productivity and indexes energetic contributions of amphibian populations to aquatic and terrestrial components of forest ecosystems [47, 48]. Understanding how buffer width relates to body size and condition can provide important insights into the indirect pathways by which forestry affects amphibian populations [49]. Similarly, knowing how adult biomass changes in response to buffer width can help clarify how cutting influences ecosystem energy flows. Previous research suggests that forest cutting is associated with reduced amphibian size and body condition, but studies examining such indirect forestry effects are relatively rare, were not conducted in buffered landscapes, and produced results that were inconsistent across species and age classes (e.g., [50–52]). Nonetheless, we expected that clearcutting would exert negative effects on amphibian body size and condition and adult biomass in our landscape, but that buffers would mitigate these effects. Because narrow buffers provide less forest habitat than wide buffers, we specifically predicted that as buffer width decreased across experimental treatments, the following characteristics of spotted-salamander and wood-frog breeding populations would also decrease:

1. individual-adult length, mass, and body condition; and
2. total breeding-adult biomass.

## Methods

### Study Site, Treatments, and Sampling

We conducted this research in a 700 km^2^ area of Hancock and Washington counties, Maine, USA (45°0’52”N, 44°48”32”N; 68°28’11”W, 67°53’10”W). Our entire study site was located in an industrial forest in Maine’s northern-interior climate zone and at the northern limit of the Downeast Ecoregion [53]. While micro-climate and micro-topographical conditions varied slightly across the site, all of our study pools were subjected to similar overall climatic and land-use conditions. For detailed descriptions of the site, experimental design, and sampling methods, see [35].

All pools were fish-free. Amphibian species composition was similar across pools, with the following species occurring at all pools: *Ambystoma maculatum*, *Lithobates sylvaticus*, *Notophathalmus viridescens*, *Lithobates clamitans*, *Pseudacris crucifer*, *Lithobates catesbeianus*, and *Lithobates palustris*. *Ambystoma laterale* and *Anaxyrus americanus* were present at all but two and one of the pools, respectively. Three additional species were rarely trapped during the experiment; these included: *Desmognathus fuscus* (a stream salamander; one individual), *Hyla versicolor* (23 individuals across seven wetlands), and *Lithobates septentrionalis* (11 individuals across six wetlands). Abiotic conditions were also similar across pools and compared to other woodland pools in the region (Table 1; [54, 55]). In particular, specific conductance values were relatively low and pH levels were somewhat acidic.

**Table 1:** Mean (± SE) of vernal pool abiotic characteristics by forestry treatment^a^.

Treatment	pH^b^	Specific Conductance (μS)	Water Temperature (°C)	Depth (m)^c^
Reference	5.60 ± 0.10	18.74 ± 1.40	14.12 ± 0.74	0.94 ± 0.18
100m	5.92 ± 0.14	31.34 ± 5.65	15.39 ± 1.25	1.14 ± 0.12
30m	5.92 ± 0.06	23.32 ± 1.05	16.27 ± 0.87	1.12 ± 0.19

^a^ Forestry treatments were: reference (i.e., uncut), 100m buffer, and 30m buffer. See Fig 1.

^b^ pH, specific conductance, and water temperature were measured at each pool in May of 2007, 2008, and 2009, using an Orion model 230A pH meter and a YSI model 85 conductivity meter.

^c^ Measured as the single greatest depth in each pool across May 2007, 2008, and 2009.

**Fig 1:**
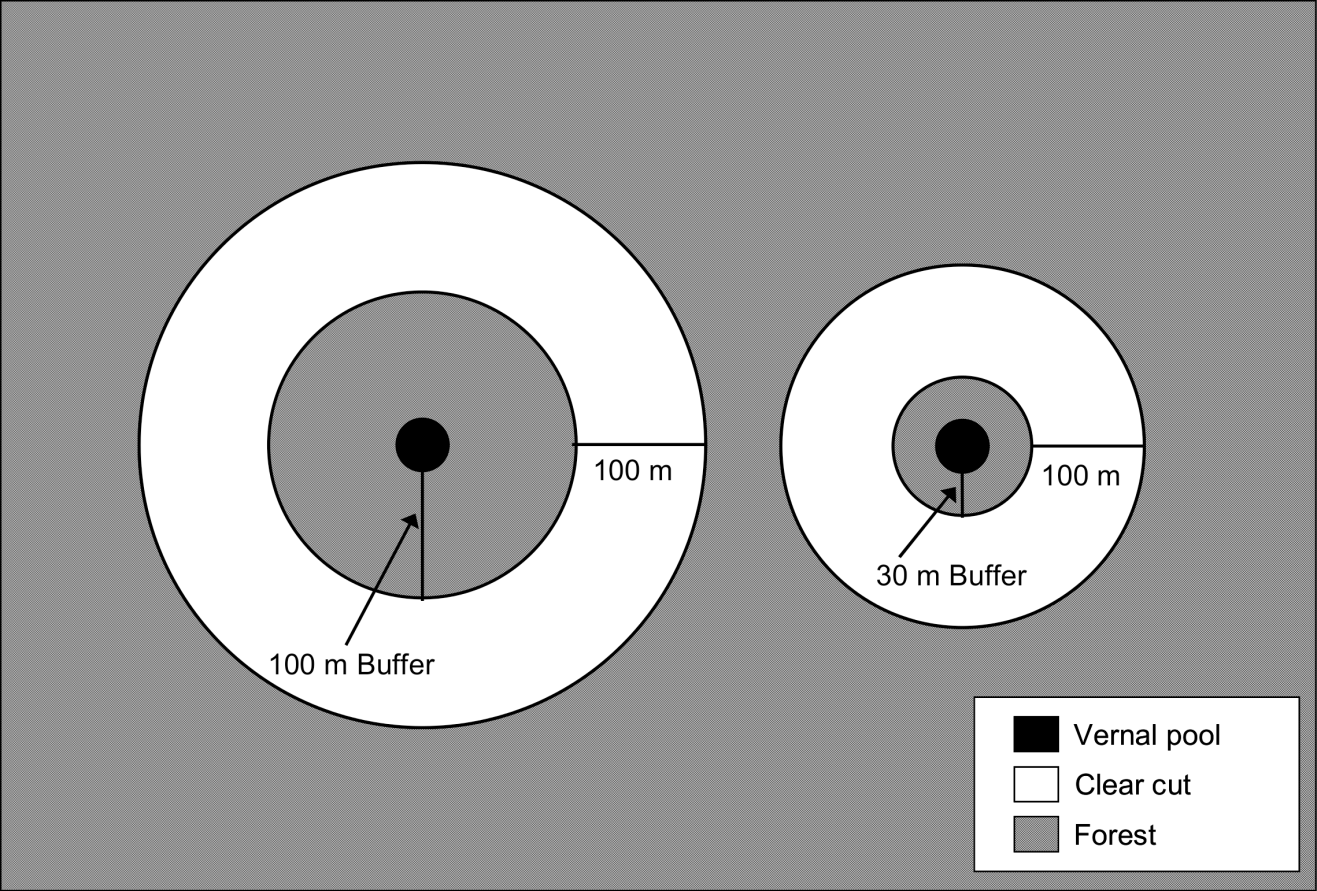
Experimental design implemented at 11 natural vernal pools in east-central Maine, USA. Undisturbed buffers of either 100m (left; n = 4) or 30m (right; n = 4) were left adjacent to pools and 100m wide clear cuts were created around the buffers. Forest beyond the clear cut was undisturbed. No cutting occurred at reference vernal pools (not shown; n = 3).

Between fall 2003 and spring 2004, the landowner created experimental buffers by clearcutting forest around the study pools. We randomly assigned each of the 11 pools to one of three treatments: reference (i.e., uncut; N = 3), 100m buffer (N = 4), or 30m buffer (N = 4). Pools in the two buffer treatments had, respectively, a 100-m or 30-m-wide upland buffer encircling the pool and a 100-m-wide concentric clearcut around the buffer (Fig 1).

In summer and fall 2003, we surrounded each of the 11 pools with a drift fence / pit fall trap array [56]. From 2004 to 2009, we opened traps in the spring after ice-out and closed traps when a pool was dry for at least seven consecutive days or in the fall when hard frosts curbed amphibian movement. We checked pitfall traps daily during periods of frequent amphibian movement (i.e., April-May and July-September) and every one to five days during periods when amphibians were less active (i.e., June and October-early November). For 2009, we did not open traps at one 30m-buffer pool because the pool was inaccessible. Our analysis is robust to this missing data, however [57].

Using the pitfall traps, we captured, counted, and sexed all adult spotted salamanders and wood frogs exiting the pools. For each individual, we also measured snout-vent or snout-urostyle length (hereafter SVL) and mass. To distinguish recaptures from new-captures and minimize the chances of counting the same individual more than once a year, we marked all exiting adults with a pool-specific toe-clip [58]. For any individual that returned to a pool the same year it was toe-clipped, we only analyzed data from its first visit. For more information on the rationale behind, and potential limitations of, this marking method, see [35]. Post-processing, we released each animal on the opposite side of the fence from which we captured it.

We measured hydroperiod for each pool in each year as the number of days the pool held water between ice-out (i.e., < 75% of the pool was covered in ice) and the day the pool dried completely. To facilitate analyses, we assigned a hydroperiod end date of October 28^th^ to pools that did not dry in a given year. We used this date because these pools still held water on this date, but it was late enough in the year that most amphibians at our study pools were inactive.

### Statistical Analyses

To test the relative impacts of buffer treatment and hydroperiod on several measures of breeding-amphibian body condition and biomass, we conducted linear mixed effects regressions (LME) using the “lme” function in S-Plus 8.0 (Insightful Corporation, Seattle, WA, USA). We defined our study population as all adults that migrated to a pool and attempted to breed in a given year. Our results thus apply to a subset of each species’ total local population and do not account for adult salamanders that skipped breeding in a given year or juveniles. For the rest of this paper, we refer to our study population as the “breeding” population.

We assessed body condition using three size metrics: SVL, mass, and a body-condition index (BCI). We used the BCI as a relative measure of energy reserves. We calculated the BCI as the residuals of an ordinary least-squares regression of mass on SVL. To obtain normal residuals for the BCI, we square-root transformed the mass and SVL data for salamanders and log-transformed mass and SVL for frogs. We calculated separate BCIs for each sex within each species. Residual-based condition indices are an appropriate tool for our study for the following reasons. First, by calculating separate BCIs for each sex within each species, we avoided the scaling issues that result when comparing BCIs across groups known to differ in size due to heterauxesis and allomorphosis [59]. Second, after transformation, our data did not violate the critical, testable assumptions inherent to BCI analysis, namely: mass and SVL were linearly related, BCI was independent of SVL, and SVL is a reliable indicator of structural size [60–62]. Finally, residual-based condition indices outperform similar measures of condition and accurately parallel energy reserves in a variety of species [61–63].

We calculated biomass as the sum of the mass of all individuals, with separate biomasses calculated for each species and each sex at each wetland in each year. For each individual counted, but not weighed (N = 328 and 748 [or 9% and 11%], for spotted salamanders and wood frogs, respectively), we assigned a mass equivalent to the imputed mean mass for its respective category. We could not determine the sex of 22 spotted salamanders and 27 wood frogs that we found dead in traps. We did not use dead individuals in the biomass analysis. To meet the assumptions of LME, we used ln(biomass + 0.5) as the y variable in all biomass analyses, except for recaptured male spotted salamanders, for which we used the untransformed biomass.

Our predictor variables were: buffer treatment, mean-pool hydroperiod (i.e., the mean hydroperiod for each pool across the six study years), standard deviation of pool hydroperiod (calculated for each pool across the six study years), an interaction between treatment and mean-pool hydroperiod, and a pair of numeric dummy variables representing an interaction between treatment and study year. We used the first dummy variable (cut.year) to distinguish whether a pool was clearcut or not. We used the second dummy variable (30m.year) to indicate marginal impacts to 30m buffer pools. This treatment X year interaction allowed us to evaluate whether impacts to the cut treatments recovered with time. We defined ‘recover’ as: being restored to values similar to those in the reference treatment, after deviating from reference-treatment values at some prior time.

We performed separate regressions for each combination of capture status (i.e., new-capture or recapture) and sex, within each species, for a total of eight regression models per size metric. We treated year and pool ID as crossed random effects [57] in all models, except when the model would not converge with crossed effects, in which case we simplified the model to include either a random intercept for year or for wetland, whichever provided a better model fit, as determined by likelihood ratio tests (LRTs). Among the simplified models, we used year random intercepts for the SVL of new-captured and recaptured male wood frogs, the BCI of recaptured female wood frogs, and the BCI of male and female recaptured spotted salamanders. Similarly, we used wetland random intercepts for the BCI of new-captured male wood frogs. We also modeled the variance-covariance structure for each regression to account for heterogeneous variance across groups and correlation among individuals from the same wetland (S1 Appendix). We used LRTs to optimize the variance-covariance structure of each model, ANOVAs to assess the overall significance of each fixed effect, and t tests to determine the significance of different treatment levels (α = 0.05). We used treatment contrasts to compare the reference treatment to each respective cut treatment (i.e., by default, there was no direct comparison between the 100m and 30m treatments; [57]). Based on an a priori decision, when the hydroperiod interaction was not significant, we removed this interaction from the model and refit the model for the remaining fixed effects. In their final forms, all models satisfied the assumptions of LME. See [35] for further details on the dummy variables used in the year X treatment interaction and the model-selection process.

### Ethics and Data Deposition Statements

We conducted all of the research in accordance with the rules of the Institutional Animal Care and Use Committee at the University of New Hampshire (IACUC-UNH). IACUC-UNH approved our research protocol, as detailed in permits: 020601 and 050604. None of the captured species were protected or endangered under federal or state law. We conducted the research on private land, with permission from the landowner. For these reasons, no additional permits or permission were needed to conduct this work. The data used in this study are available from the Dryad database (http://dx.doi.org/10.5061/dryad.62ks6).

## Results

Over the six study years, the 11 vernal pools produced over 47 kg of breeding spotted salamanders and 64 kg of breeding wood frogs. This biomass represented 3624 breeding spotted salamanders and 6521 breeding wood frogs. Descriptive statistics are provided in Table 2 for size and body condition and in Table 3 for biomass.

**Table 2:** Mean and variability of predictor and amphibian size variables, by species, capture status, sex, and forestry treatment.

			**Mean ± SE**	**Range**				
**Mean hydroperiod (days)**			126.0±6.0	44.8–197.0				
**SD hydroperiod** ^**a**^ **(days)**			31.8±1.6	6.3–48.8				
			**SVL/SUL** ^**b**^ **(mm)**		**Mass (g)**		**BCI** ^**c**^	
			**Mean ± SE**	**Range**	**Mean ± SE**	**Range**	**Mean ± SE**	**Range**
**Spotted Salamander**								
recapture	F	Reference	82.4±1.0	67.0–99.0	18.1±0.5	8.1–26.0	0.204±0.042	-0.637–1.082
		100m	82.6±0.5	61.0–100.0	17.4±0.2	7.3–28.0	0.098±0.024	-0.942–0.912
		30m	75.0±0.7	55.0–90.0	13.1±0.3	6.5–22.9	-0.096±0.035	-0.794–0.580
	M	Reference	74.8±0.6	60.0–90.0	14.0±0.3	8.9–21.0	0.242±0.027	-0.514–1.034
		100m	73.1±0.4	54.0–98.0	12.8±0.1	7.3–21.0	0.146±0.018	-0.715–1.009
		30m	67.1±0.6	51.0–85.0	9.8±0.2	5.5–20.5	-0.040±0.030	-0.685–0.849
new-capture	F	Reference	82.3±0.6	61.0–101.0	17.3±0.3	7.5–31.0	0.094±0.026	-0.883–1.188
		100m	81.7±0.3	53.0–102.0	16.5±0.1	7.1–26.9	0.032±0.015	-0.979–1.939
		30m	74.8±0.4	53.0–95.0	12.7±0.2	6.0–25.0	-0.157±0.017	-1.153–0.962
	M	Reference	73.6±0.5	55.0–96.0	12.3±0.2	5.7–22.2	0.053±0.020	-0.638–0.825
		100m	72.5±0.3	52.0–96.0	11.6±0.1	5.3–24.0	-0.013±0.012	-1.589–1.103
		30m	65.4±0.3	51.0–95.0	8.9±0.1	4.5–18.9	-0.128±0.011	-0.742–0.719
**Wood Frog**										
recapture	F	Reference	51.9±0.3	44.0–58.0	13.1±0.2	8.8–18.0	0.031±0.013	-0.318–0.434
		100m	51.0±0.4	35.0–59.0	12.8±0.2	6.6–19.3	0.044±0.014	-0.313–0.454
		30m	49.8±0.5	35.0–56.0	11.0±0.3	5.3–17.8	-0.045±0.021	-0.243–0.445
	M	Reference	44.1±0.2	31.0–56.0	9.1±0.1	4.8–14.0	0.020±0.008	-0.462–0.488
		100m	44.5±0.2	31.0–54.0	9.2±0.1	4.1–13.4	0.020±0.010	-0.409–0.710
		30m	43.4±0.3	31.0–56.0	8.8±0.2	5.5–14.3	0.011±0.015	-0.273–0.542
new-capture	F	Reference	49.9±0.1	33.0–60.0	12.1±0.1	3.7–20.8	0.011±0.006	-0.915–0.656
		100m	49.6±0.2	37.0–60.0	12.1±0.1	5.0–22.0	0.022±0.006	-0.518–0.555
		30m	48.6±0.2	35.0–59.0	10.9±0.1	3.8–21.5	-0.043±0.007	-0.958–0.606
	M	Reference	44.1±0.1	33.0–55.0	9.0±0.1	3.3–19.0	0.014±0.004	-0.777–0.635
		100m	43.0±0.1	30.0–61.0	8.7±0.1	4.2–19.6	0.005±0.005	-0.729–0.821
		30m	42.6±0.1	27.0–53.0	8.3±0.1	4.1–13.8	-0.033±0.005	-0.638–0.611

^a^ Standard deviation of pool hydroperiod.

^b^ Snout-vent or snout-urodyle length.

^c^ Body condition index. Obtained via ordinary least squares regression of mass on SVL/SUL. Mass and SVL/SUL were square-root transformed for salamanders and log-transformed for frogs, prior to regression. BCI measures relative energy reserves. BCI > 0 indicates better body condition than BCI < 0. Mean BCI may not equal zero because BCI was calculated over recaptured and new-captured animals combined, for each sex.

**Table 3:** Mean and variability of total annual breeding amphibian biomass by species, forestry treatment, capture status, and sex.

	Adult Biomass (g)				
Species	Sex	Treatment	Mean ± SE	Range	Total
**Spotted Salamander**					
recapture	F	Reference	71.2±17.2	0–227.2	1068.4
		100m	160.0±66.5	0–1273.4	3199.1
		30m	52.2±14.6	0–240.5	992.3
	M	Reference	102.6±23.1	0–286.0	1539.5
		100m	171.8±48.8	0–750.8	3435.9
		30m	55.7±12.0	0–191.6	1057.8
new-capture	F	Reference	214.8±26.1	29.6–436.0	3866.9
		100m	390.5±108.9	0–2158.6	9373.1
		30m	213.9±32.0	27.4–551.5	4920.4
	M	Reference	179.6±33.1	0–570.9	3232.9
		100m	383.1±96.7	0–1493.5	9195.4
		30m	210.8±37.2	45.0–819.7	4848.7
**Wood Frog**					
recapture	F	Reference	99.3±25.7	0–337.8	1489.2
		100m	74.9±27.1	0–515.4	1498.8
		30m	33.9±11.0	0–175.2	645.0
	M	Reference	222.1±82.6	26.3–1347.7	3331.4
		100m	106.9±25.9	0–421.6	2137.5
		30m	55.5±11.5	0–189.8	1054.4
new-capture	F	Reference	498.2±87.5	109.8–1319.2	8968.2
		100m	341.3±55.2	17.8–1049.5	8192.3
		30m	329.1±48.9	22.7–897.5	7570.2
	M	Reference	600.7±139.8	88.2–2765.9	10812.0
		100m	385.4±69.6	27.5–1386.3	9249.4
		30m	390.1±50.5	51.8–855.2	8972.3

### Spotted Salamanders

In general, we found that spotted salamanders were smaller and had worse body condition at 30m, compared to reference, pools. For some, but not all, combinations of capture status, sex, and size metric, we observed partial recovery of the size metric at 30m-buffer pools over the six study years. We found less consistent relationships between treatment and biomass than between treatment and body size/condition. All results presented in this section were statistically significant, unless otherwise indicated.

**Fig 2:**
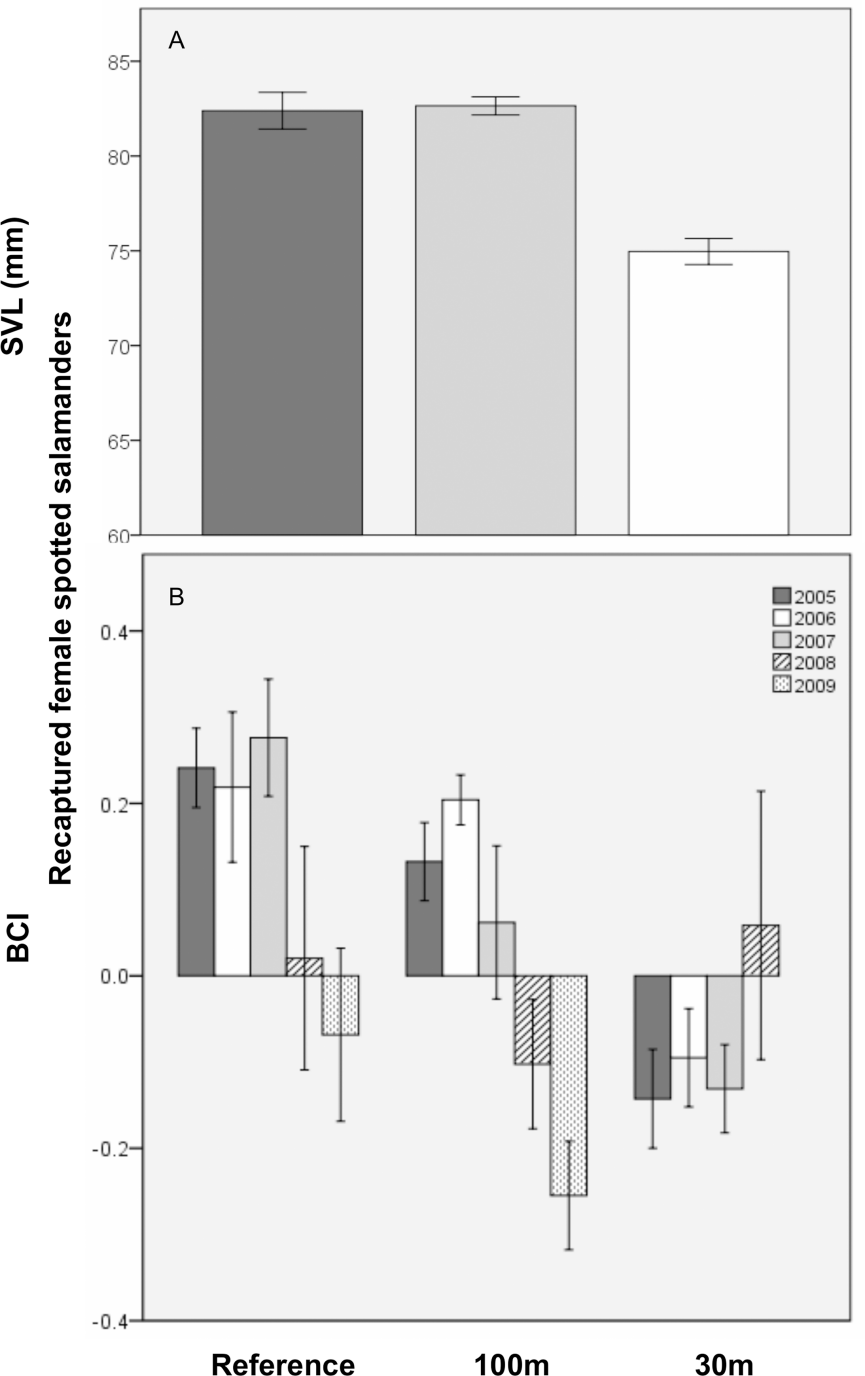
Mean (±1SE) size of recaptured breeding female spotted salamanders at 11 vernal pools in east-central Maine, USA. A) Snout-vent length (SVL; mm) across three experimental forestry treatments and B) body condition index (BCI) by forestry treatment and study year. Treatments were: reference (uncut), 100m undisturbed buffer, and 30m undisturbed buffer.

Recaptured female salamanders were, throughout the study and on average, predicted to be 9.1 mm shorter at 30m versus reference pools (Table 4; Fig 2). (Note: no females were recaptured at 30m-buffer pools in 2009). Similarly, in the first recapture year (i.e., 2005), the average recaptured female at the 30m-buffer pools was predicted to weigh 7 g less, and have worse body condition, than her reference-pool counterpart. However, mass and BCI were both predicted to recover to mean reference levels by about 9.5 years post-cut. Conversely, recaptured-female body condition at the 100m-buffer pools worsened with time, so that by the study’s end, 100m-pool BCI was predicted to be about two times lower than the mean reference BCI. BCI also decreased, in all treatments, with increasing hydroperiod duration and variability. Additionally, recaptured female biomass was predicted to decrease by about 58% per year at 30m-buffer pools, but tended to increase (i.e., was marginally significant) by about 2.4% per each additional day of mean hydroperiod in all treatments. Finally, SVL, mass, and biomass did not differ significantly between the 100m and reference treatments.

**Table 4:** Linear mixed regression results showing the relative impact of forestry treatment, hydroperiod, and study year on size, body condition, and total annual biomass of breeding spotted salamanders and wood frogs.

Size Metric	Predictor^c^	F value_(df)_ ^j^	t value_(df)_ ^l^	Coefficient ± SE
**Spotted Salamander**				
**Recaptured Females**				
**SVL** ^**a**^ **(mm)**	treatment(30m)^d^	3.61_(2,312)_ *	-2.10_(312)_ *	-9.089±4.336
	intercept	683.04_(1,312)_ ***	26.13_(312)_ ***	86.708±3.318
**mass (g)**	treatment(30m)	7.90_(2,6)_ *	-3.89_(6)_ *	-8.938±2.296
	30m.year^e^	4.28_(1,300)_ *	2.07_(300)_ *	1.049±0.507
	intercept	64.15_(1,300)_ ***	8.01_(300)_ ***	20.062±2.505
**BCI** ^**b**^	treatment(30m)	6.38_(2,301)_ *	-2.01_(301)_ *	-0.385±0.192
	cut.year^f^	5.78_(1,301)_ *	-2.40_(301)_ *	-0.066±0.027
	30m.year	4.28_(1,301)_ *	2.07_(301)_ *	0.105±0.051
	mean.hydro^g^	7.91_(1,301)_ *	-2.81_(301)_ *	-0.002±0.001
	sd.hydro^h^	7.46_(1,301)_ *	-2.73_(301)_ *	-0.005±0.002
	intercept	15.25_(1,301)_ **	3.91_(301)_ **	0.530±0.136
**biomass (g)**	30m.year	16.37_(1,47)_ **	-4.05_(47)_ **	-0.734±0.181
	mean.hydro	4.00_(1,47)_ ^•^	2.00_(47)_ ^•^	0.024±0.012
**New-captured Females**				
**SVL (mm)**	treatment(30m)	4.78_(2,1079)_ *	-2.53_(1079)_ *	-7.820±3.095
	30m.year	5.75_(1,1079)_ *	2.40_(1079)_ *	0.660±0.275
	intercept	397.14_(1,1079)_ ***	19.93_(1079)_ ***	84.855±4.258
**mass (g)**	treatment(30m)	3.25_(2,1051)_ *	-2.34_(1051)_ *	-4.461±1.905
	intercept	51.52_(1,1051)_ ***	7.18_(1051)_ ***	19.008±2.648
**BCI**	treatment(30m)	2.34_(2,1054)_ ^•^	-1.96_(1054)_ ^•^	-0.229±0.117
**biomass (g)**	treatment(100m)^i^ *mean.hydro	7.91_(2,45)_ *	3.88_(45)_ **	0.040±0.013
	treatment(100m)	6.62_(2,45)_ *	-3.48_(45)_ *	-5.212±1.496
	intercept	21.21_(1,45)_ ***	4.61_(45)_ ***	5.564±1.208
**Recaptured Males**				
**SVL (mm)**	treatment(30m)	5.38_(2,478)_ *	-3.05_(478)_ *	-9.778±3.201
	intercept	415.79_(1,478)_ ***	20.39_(478)_ ***	79.218±3.885
**mass (g)**	treatment(30m)	7.31_(2,473)_ **	-3.53_(473)_ **	-4.796±1.359
	30m.year	3.05_(1,473)_ ^•^	1.75_(473)_ ^•^	0.373±0.214
	intercept	92.18_(1,473)_ ***	9.60_(473)_ ***	15.321±1.596
**BCI**	treatment(30m)	5.15_(2,468)_ *	-3.17_(468)_ *	-0.439±0.138
	mean.hydro	5.54_(1,468)_ *	-2.35_(468)_ *	-0.001±<0.001
	intercept	6.73_(1,468)_ *	2.59_(468)_ *	0.297±0.114
**biomass (g)**	treatment(100m)*mean.hydro	4.24_(2,45)_ *	2.90_(45)_ *	3.038±1.047
	treatment(100m)	3.15_(2,45)_ ^•^	-2.50_(45)_ *	-314.310±125.594
	sd.hydro	3.62_(1,45)_ ^•^	-1.90_(45)_ ^•^	-3.457±1.818
	intercept	4.19_(1,45)_ *	2.05_(45)_ *	251.847±123.053
**New-captured Males**				
**SVL (mm)**	treatment(30m)	4.36_(2,1444)_ *	-2.73_(1444)_ *	-7.820±2.865
	cut.year	3.15_(1,1444)_ ^•^	1.77_(1444)_ ^•^	0.500±0.282
	30m.year	5.39_(1,1444)_ *	2.32_(1444)_ *	0.556±0.239
	intercept	398.02_(1,1444)_ ***	19.95_(1444)_ ***	77.363±3.878
**mass (g)**	treatment(30m)	6.78_(2,1410)_ *	-3.01_(1410)_ *	-3.620±1.204
	30m.year	22.70_(1,1410)_ ***	4.76_(1410)_ ***	0.409±0.086
	mean.hydro	3.27_(1,1410)_ ^•^	-1.81_(1410)_ ^•^	-0.015±0.008
	intercept	78.92_(1,1410)_ ***	8.88_(1410)_ ***	14.336±1.614
**BCI**	treatment(30m)	11.02_(2,1410)_ ***	-3.22_(1410)_ *	-0.274±0.085
	30m.year	12.03_(1,1410)_ **	3.47_(1410)_ **	0.043±0.012
	mean.hydro	3.62_(1,1410)_ ^•^	-1.90_(1410)_ ^•^	-0.001±<0.001
**biomass (g)**	treatment(100m)*mean.hydro	4.28_(2,45)_ *	2.91_(45)_ *	0.030±0.013
	treatment(100m)	4.53_(2,45)_ *	-2.90_(45)_ *	-4.409±1.522
	cut.year	3.79_(1,45)_ ^•^	1.95_(45)_ ^•^	0.172±0.088
	30m.year	9.45_(1,45)_ *	-3.07_(45)_ *	-0.269±0.088
	intercept	16.56_(1,45)_ **	4.07_(45)_ **	5.202±1.278
**Wood Frogs**				
**Recaptured Females**				
**SUL** ^**a**^ **(mm)**	30m.year	4.21_(1,284)_ *	-2.05_(284)_ *	-1.284±0.626
	intercept	473.14_(1,284)_ ***	21.75_(284)_ ***	53.190±2.445
**mass (g)**	intercept	89.78_(1,236)_ ***	9.48 _(236)_ ***	14.303±1.509
**BCI**	treatment(30m)	4.44_(2, 231)_ *	-2.97_(231)_ *	-0.221±0.074
	sd.hydro	5.97_(1,231)_ *	-2.44_(231)_ *	-0.002±0.001
**biomass (g)**	treatment(100m)*mean.hydro	6.10_(2,45)_ *	3.49_(45_)*	0.038±0.011
	treatment(100m)	5.94_(2,45)_ *	-3.33_(45)_ *	-5.794±1.741
	treatment(30m)		-1.89_(45)_ ^•^	-5.005±2.654
	intercept	14.18_(1,45)_ **	3.77_(45)_ **	4.958±1.317
**New-captured Females**				
**SUL (mm)**	cut.year	5.50_(1,2041)_ *	2.34_(2041)_ *	0.349±0.149
	intercept	1019.22_(1,2040)_ ***	31.93_(2041)_ ***	50.362±1.577
**mass (g)**	cut.year	5.55_(1,1869)_ *	2.36_(1869)_ *	0.229±0.097
	intercept	116.32_(1,1869)_ ***	10.78_(1869)_ ***	12.572±1.166
**BCI**		ns^k^		
**biomass (g)**	treatment(100m)*mean.hydro	3.45_(2,56)_ *	2.63_(56)_ *	0.015±0.006
	treatment(100m)	5.98_(2,56)_ *	-3.45_(56)_ *	-2.931±0.850
	30m.year	5.58_(1,56)_ *	-2.36_(56)_ *	-0.268±0.113
	intercept	59.07_(1,56)_ ***	7.69_(56)_ ***	5.737±0.746
**Recaptured Males**				
**SUL (mm)**	30m.year	3.91_(1,706)_ *	-1.98_(706)_ *	-0.914±0.462
	sd.hydro	11.44_(1,706)_ **	-3.38_(706)_ **	-0.045±0.013
	intercept	2064.37_(1,706)_ ***	45.44_(706)_ ***	44.803±0.986
**mass (g)**	sd.hydro	6.35_(1,684)_ *	-2.52_(684)_ *	-0.033±0.013
	intercept	202.99_(1,684)_ ***	14.25_(684)_ ***	9.559±0.671
**BCI**	sd.hydro	2.90_(1,685)_ ^•^	-1.70_(685)_ ^•^	-0.002±0.001
**biomass (g)**	30m.year	3.50_(1,47)_ ^•^	-1.87_(47)_ ^•^	-0.518±0.277
	mean.hydro	7.11_(1,47)_ *	2.67_(47)_ *	0.015±0.006
	intercept	5.21_(1,47)_ *	2.28_(47)_ *	2.471±1.083
**New-captured Males**				
**SUL (mm)**	intercept	5523.34_(1,3082)_ ***	4.32_(3082)_ ***	44.060±0.593
**mass (g)**	sd.hydro	5.86_(1,2932)_ *	-2.42_(2932)_ *	-0.022±0.009
	intercept	432.10_(1,2932)_ ***	0.79_(2932)_ ***	9.403±0.452
**BCI**	sd.hydro	5.60_(1,6)_ ^•^	-2.37_(6)_ ^•^	-0.002±0.001
**biomass (g)**	mean.hydro	5.11_(1,58)_ *	2.26_(58)_ *	0.008±0.004
	intercept	43.91_(1,58)_ ***	6.63_(58)_ ***	4.775±0.721

^a^ SVL = snout-vent length; SUL = snout-urodyle length.

^b^ Body condition index. BCI > 0 indicates better body condition than BCI < 0.

^c^ All models included the following predictors: treatment, mean pool hydroperiod, standard deviation of pool hydroperiod, a treatmentXyear interaction, and a treatmentXmean.hydro interaction. Based on an a priori decision, we dropped the treatmentXmean.hydro interaction from the model if the interaction was not significant. Only significant fixed-effect results are shown.

^d^ Categorical variable, coded 0 = reference treatment and 1 = 30m treatment.

^e^ Dummy variable representing the marginal impact of the 30m treatment over the six study years.

^f^ Dummy variable representing the difference between the reference treatment and the two cut treatments, over the six study years.

^g^ Mean pool hydroperiod in days.

^h^ Standard deviation of pool hydroperiod in days.

^i^ Categorical variable, coded 0 = reference treatment and 1 = 100m treatment.

^j^ We used F tests to assess overall significance of each variable. We provide results just once for each categorical variable.

^k^ None of the independent variables were significant predictors of female new-capture wood frog body condition.

^l^ We used t tests to compare between individual levels of categorical predictors.

*** p < 0.0001

** p < 0.001

* p < 0.05

^•^ 0.05 ≤ p <0.1

**Fig 3:**
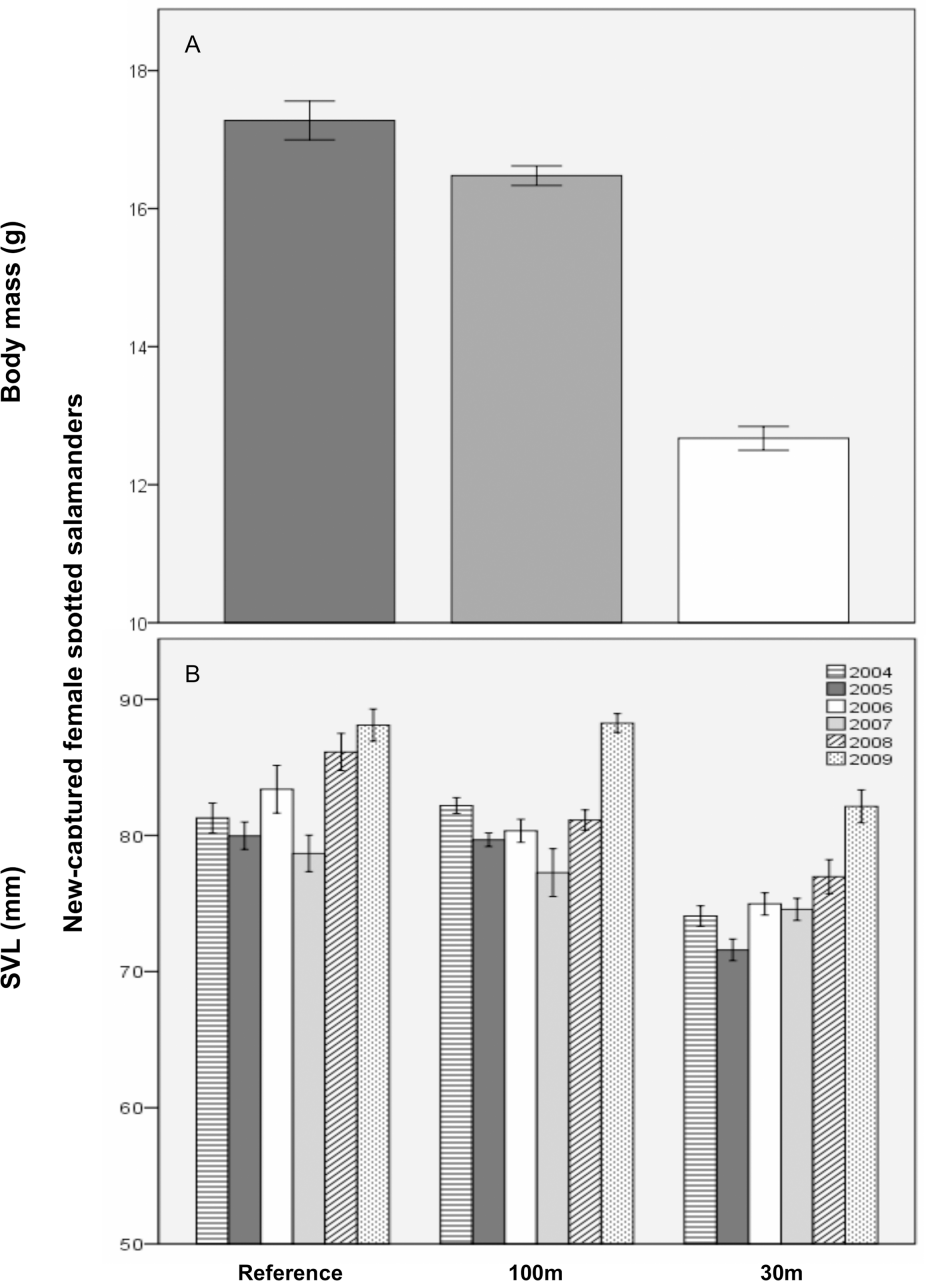
Mean (±1SE) size of new-captured breeding female spotted salamanders at 11 vernal pools in east-central Maine, USA. A) Body mass (g) across three experimental forestry treatments and B) snout-vent length (SVL; mm) by forestry treatment and study year. Treatments were: reference (uncut), 100m undisturbed buffer, 30m undisturbed buffer.

New-captured female spotted salamanders were predicted to weigh, on average and for the duration of the study, 4.5 g less at 30m-buffer pools than at reference pools (Fig 3). They also tended to have persistently worse body condition at 30m-buffer pools. During the first year post-cut, new-captured females were predicted to be, on average, 7.3 mm shorter in the 30m versus reference treatment. SVL at 30m-buffer pools was predicted to recover to mean reference levels by about 14 years post-cut. For new-captured female biomass, the 30m and reference treatments did not differ, but 100m-treatment biomass depended on mean pool hydroperiod. Short-hydroperiod pools were predicted to produce much lower biomass in the 100m, compared to the reference, treatment. For each additional day of mean hydroperiod, however, biomass at the 100m-buffer pools was predicted to increase by about 3.8%. Finally, SVL, mass, and BCI did not differ significantly between the 100m and reference treatments.

For recaptured male spotted salamanders, both SVL and BCI were lower at 30m-buffer pools than reference pools and failed to recover to reference levels. On average, recaptured males were predicted to be 9.8 mm shorter at 30m-buffer pools. During the first recapture year, recaptured males were also predicted to weigh, on average, about 4 g less at 30m versus reference pools. A marginally significant 30mXyear interaction suggests recaptured male mass would take about 11 years to recover to mean reference levels. Recaptured male body condition and biomass were also influenced by hydroperiod. For all treatments, body condition declined with increasing mean hydroperiod and pools with more variable hydroperiod tended to support lower total biomass. For short-hydroperiod pools, we also found less biomass in the 100m versus the reference treatment, but the associated coefficient and standard error were quite large and should be cautiously interpreted. Nevertheless, for each additional day of mean hydroperiod, 100m biomass was predicted to increase by 2.5 g. Finally, SVL, mass and BCI did not differ significantly between the 100m and reference treatments.

**Fig 4:**
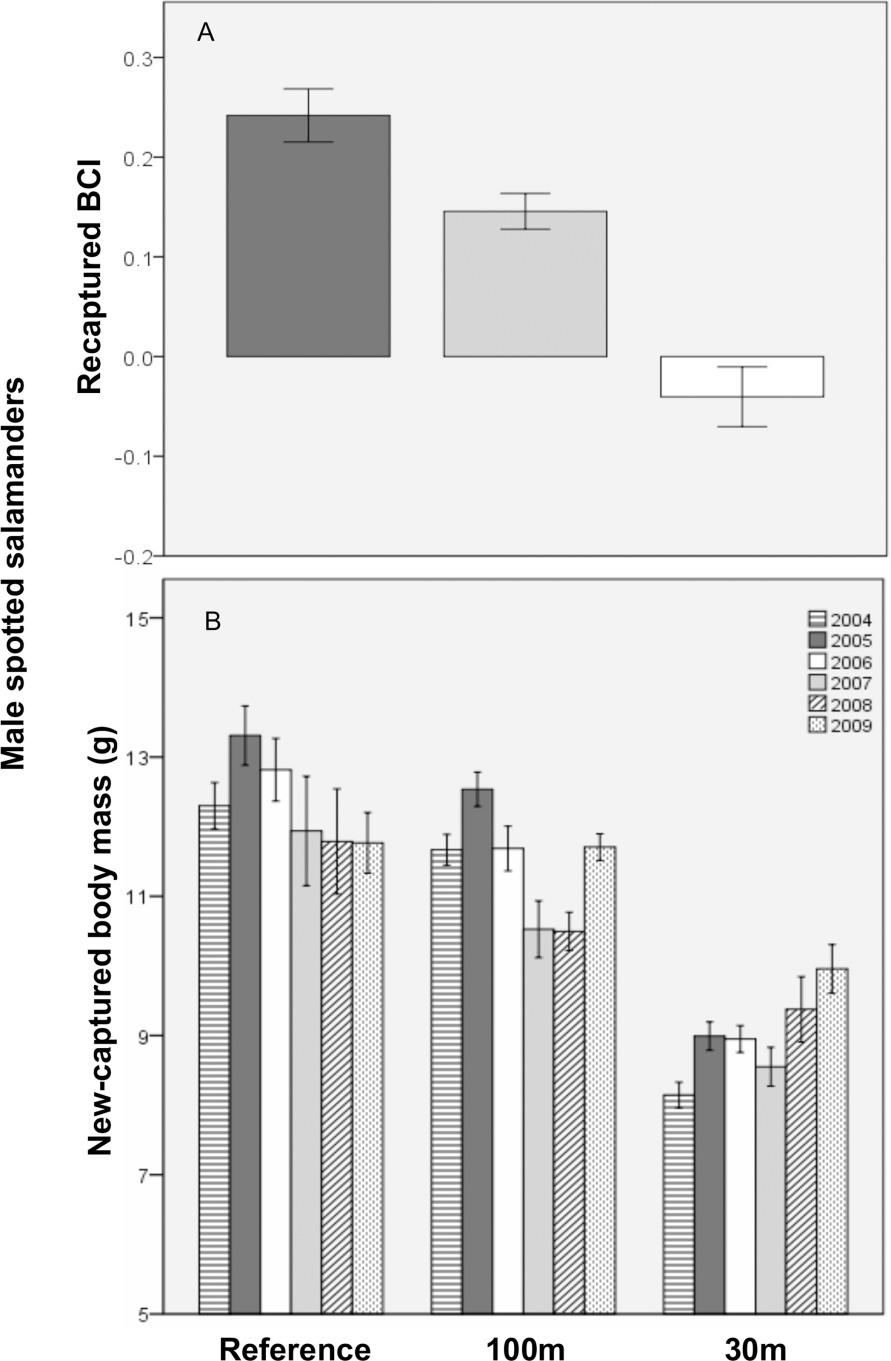
Mean (±1SE) size of breeding male spotted salamanders at 11 vernal pools in east-central Maine, USA. A) Body condition index (BCI) of recaptured males across three experimental forestry treatments and B) body mass (g) of new-captured males by forestry treatment and study year. Treatments were: reference (uncut), 100m undisturbed buffer, and 30m undisturbed buffer.

New-captured male spotted salamanders were predicted, on average, to be 6.8 mm shorter, weigh 3.2 g less, and have worse body condition, at 30m-buffer pools than at reference pools during the first year post-cut (Fig 4). However, all three size metrics were predicted to recover with time at the 30m-buffer pools. The predicted recovery periods were, respectively: 8, 10, and 9 years, for SVL, mass, and BCI. Conversely, biomass at 30m-buffer pools was predicted to decrease by about 9% each year. Biomass at 100m-buffer pools depended on year and mean hydroperiod. During the first year post-cut, on average, less biomass was predicted at 100m versus reference pools. For each successive year, however, 100m biomass was predicted to increase by about 19%, so that by 3.5 years post-cut, similar amounts of biomass were predicted from typical 100m and reference pools. We also found that short-hydroperiod pools had much less biomass in the 100m versus reference treatment, but biomass increased at 100m-buffer pools by about 2.9% per additional day of mean hydroperiod. Though body mass and BCI did not differ significantly between the 100m and reference treatments, a marginally significant result indicated SVL increased by 0.5 mm per year in the 100m treatment. Finally and for all treatments, new-capture male mass and body condition tended to decrease as mean hydroperiod increased.

### Wood Frogs

For wood frog size and biomass generally, females and recaptured adults were more sensitive to buffer treatment than males and new-captured adults, respectively. Additionally, hydroperiod was a strong predictor across size metric, sex, and capture status. All results presented in this section were statistically significant, unless otherwise indicated.

**Fig 5:**
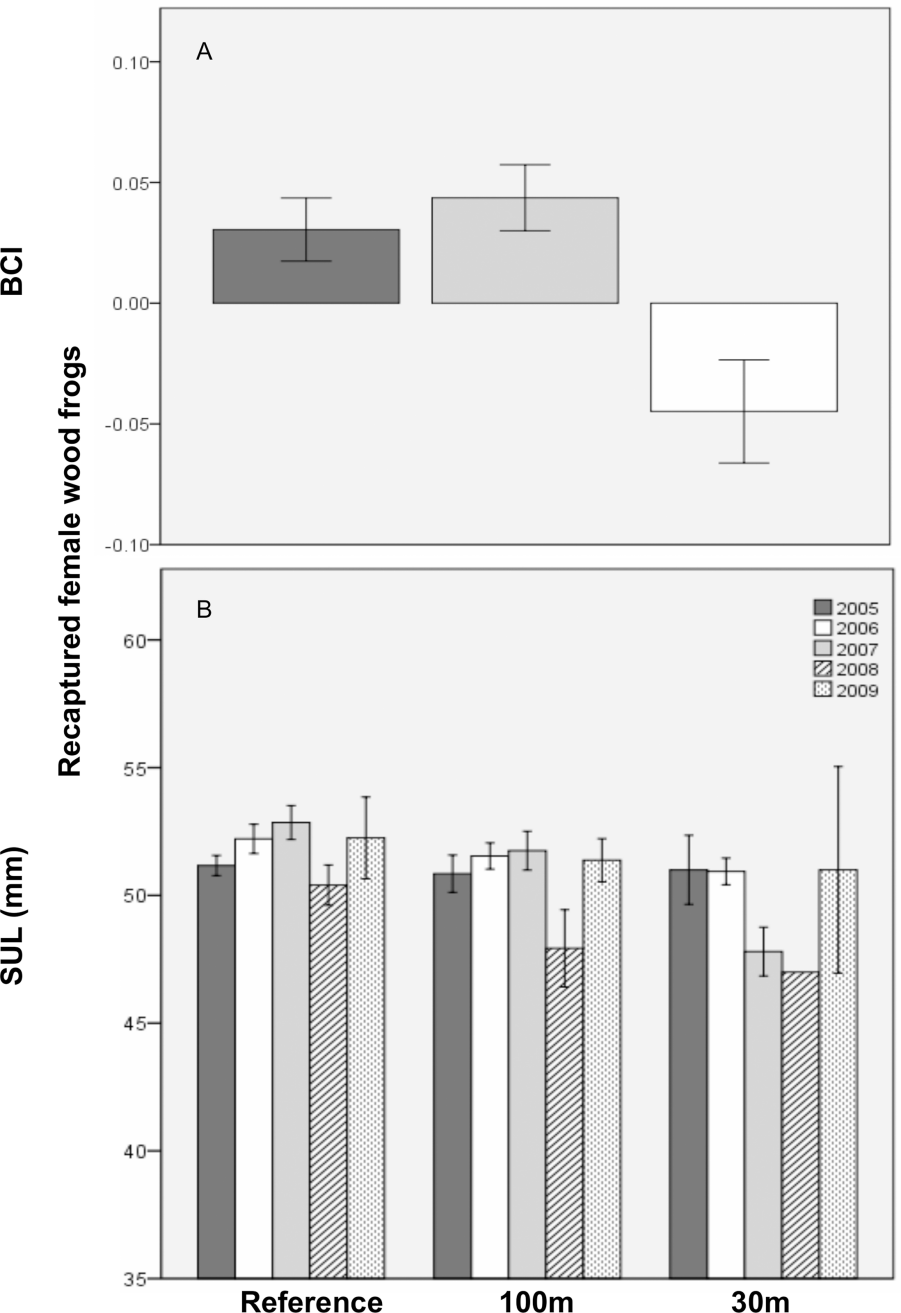
Mean (±1SE) size of recaptured breeding female wood frogs at 11 vernal pools in east-central Maine, USA. A) Body condition index (BCI) across three experimental forestry treatments and B) snout-urodyle length (SUL; mm) by forestry treatment and study year. Treatments were: reference (uncut), 100m undisturbed buffer, and 30m undisturbed buffer.

**Fig 6:**
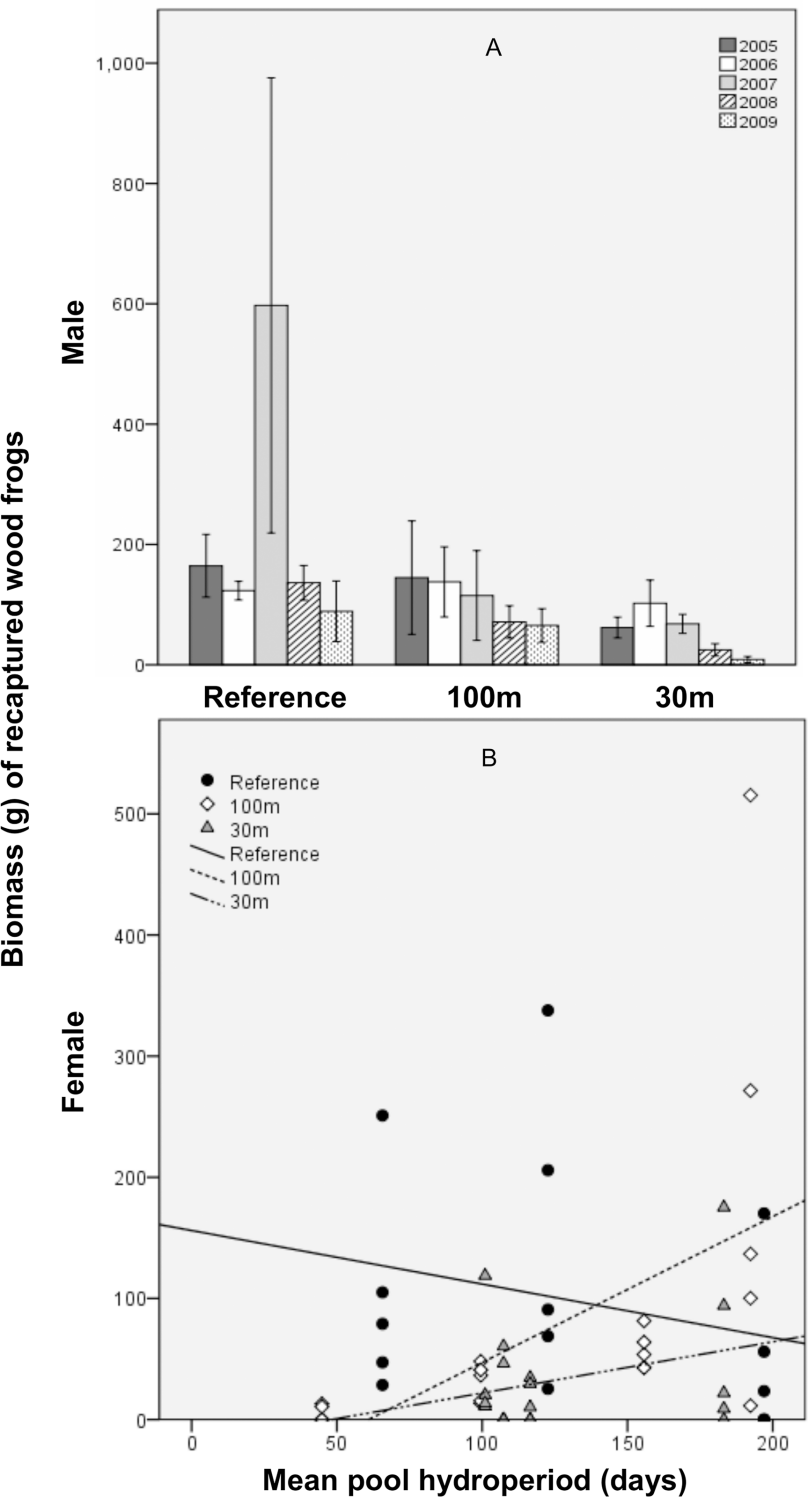
Total annual biomass (g) of recaptured breeding wood frogs at 11 vernal pools in east-central Maine, USA. A) Mean (±1SE) biomass of male frogs by forestry treatment and study year and B) biomass of female frogs by forestry treatment and mean pool hydroperiod (days). Treatments were: reference (uncut), 100m undisturbed buffer, 30m undisturbed buffer.

For recaptured female wood frogs, BCI (Fig 5) and biomass (marginally significant) were predicted to be, on average, lower at 30m-buffer pools than at reference pools, and did not recover during the study. Specifically, biomass in the 30m treatment was only about 1/3^rd^ of that in the reference treatment. Further, SVL was predicted to decrease by 1.32 mm/year at the 30m-buffer pools. At short-hydroperiod pools, biomass was lower in the 100m versus reference treatment, but for each additional day of mean hydroperiod, 100m biomass was predicted to increase by about 3% (Fig 6). Body condition worsened as hydroperiod variability increased. Finally, recaptured female mass was unrelated to treatment, year, or hydroperiod; and SVL and BCI did not differ significantly between the 100m and reference treatment.

For new-captured female wood frogs, biomass in the 30m treatment was predicted to decrease by about 14% per year. Similar to recaptured females, new-captured female biomass at short-hydroperiod pools was lower in the 100m versus reference treatment, but 100m biomass was predicted to increase with each additional day of mean hydroperiod by about 1.4%. In both cut treatments, SVL and mass were predicted to increase post-cut, by 0.3 mm/year and 0.2 g/year, respectively. Finally, BCI was unrelated to treatment, year, or hydroperiod.

**Fig 7:**
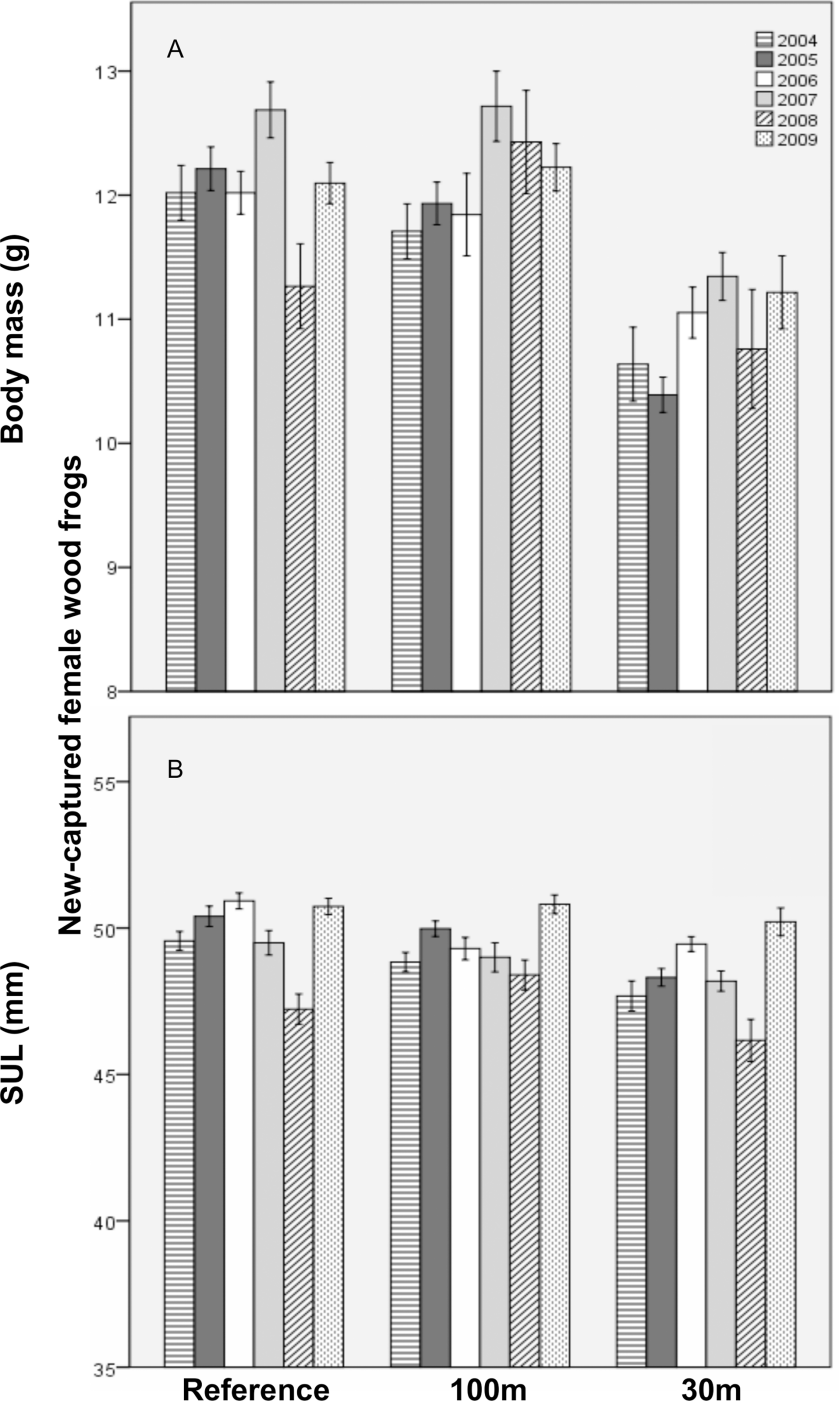
Mean (±1SE) size of new-captured breeding female wood frogs at 11 vernal pools in east-central Maine, USA. A) Body mass (g) and B) snout-urodyle length (SUL; mm) by forestry treatment and study year. Treatments were: reference (uncut), 100m undisturbed buffer, and 30m undisturbed buffer.

For recaptured male wood frogs, SVL and biomass were predicted to decrease at 30m-buffer pools by 0.9 mm/year and about 44% per year, respectively (Figs 6 and 7; the pattern was marginally significant for biomass). Similarly, but across all treatments, as hydroperiod variability increased, SVL, mass, and BCI (marginally significant) decreased, such that for each additional day of hydroperiod variability, frogs were predicted to be 0.04 mm shorter and weigh 0.03 g less. Conversely, for each additional day of mean hydroperiod, recaptured male biomass was predicted to increase by about 1.5%. Recaptured male mass and BCI were unrelated to treatment. Finally, both SVL and biomass did not differ significantly between the 100m and reference treatments.

For new-captured male wood frogs, for every additional day of hydroperiod variability, body mass was predicted to decrease by 0.03 g and BCI tended to decrease. For each additional day of mean hydroperiod duration, however, biomass was predicted to increase by about 1.5%. SVL, body mass, BCI, and biomass of new-captured males were all unrelated to treatment and year; SVL also did not differ with hydroperiod.

## Discussion

This is the first landscape-scale experiment to test how buffer width affects the impacts of forest clearcutting on amphibian body size, condition, and biomass at natural vernal pools. As hypothesized, we generally found that amphibians were smaller, had lower energy reserves, and supported less biomass at pools with a narrow 30m buffer versus 100m-buffer or reference pools. The response at 100m-buffer pools was typically mediated by hydroperiod: short-hydroperiod pools had less biomass in the 100m versus reference treatment. Overall, spotted salamanders were more affected than wood frogs and recaptured adults were more sensitive than new-captured adults. Though some size and condition metrics started to recover during the first six years post-cut, other impacts persisted or worsened. Our study demonstrates that clearcutting is associated with strong sub-lethal effects on local amphibian populations. These effects potentially signal reduced population resilience, which could alter local and regional population and community dynamics. Wider buffers helped mitigate the magnitude and duration of these effects.

### Size and Condition

#### Mechanisms

Food energy is allocated to one of four uses: maintenance, growth, reproduction, or storage. As ectotherms, amphibians have low maintenance costs and efficiently convert food to biomass [64]. Various factors can disrupt this efficiency, causing reallocation of energetic investments and reduced body size and condition. In clearcuts, high temperatures and low humidity [65–67] can elevate metabolic rates [68, 69] and maintenance costs [42, 70], while inhibiting foraging [71, 72]. Higher predation risk [12, 73] or less prey in cuts or along cut edges could also limit food intake [70, 74]. Such problems can compound if robust individuals claim prime buffer habitat, ‘despotically’ forcing stunted individuals into the cut [49, 51, 75]. Alternatively, individuals may avoid the cut by remaining in the buffer, causing overcrowding. This could limit food consumption and elevate maintenance costs, through increased competition for prey and shelter [76–78], predation risk [12], and the stress associated with competitive interactions and predator avoidance [79–81]. With increased maintenance and reduced food intake, individuals would be forced to invest less in reproduction, growth, and/or storage. Negative feedback, whereby small adults produce small eggs [39, 82], which become disadvantaged larvae [76, 83, 84], which metamorphose into stunted adults [85–87], could reinforce this pattern. Alternatively, large or well-conditioned adults might be killed during cutting or emigrate to other pools [88] leaving small, weak individuals behind.

Overall, reduced size and body condition suggest poor habitat quality in the 30m treatment [81, 89, 90]. By comparing SVL, mass, and condition, we can discern how habitat degradation altered energy allocation across treatments, species, sexes, and capture classes and start to elucidate mechanisms by which timber harvest influences amphibian populations. For recaptured spotted salamanders, SVL showed no recovery during the six study years, whereas female mass and condition were predicted to recover by about 9 years post-cut and male mass by about 11 years. Clearly, recaptured adults did not invest in structural growth, but prioritized maintenance, reproduction, and, for females, storage. These recovery trajectories suggest recaptured salamander size and condition take 10+ years after a clearcut to either rebound or adjust to reduced habitat carrying capacity [81]. Among new-captures, by contrast, the SVL of both sexes and male mass and body condition, were recovering by the experiment’s end. New-captured salamanders include immigrants and residents who previously refrained from breeding. New-capture recovery trajectories suggest several possible conclusions. First, 30m-treatment habitat alterations were least severe for male new-captures. Second, large (long) immigrants perceived the 30m treatment as viable habitat only after several years of cut regeneration. Finally, resident new-capture salamanders adopted differing allocation strategies, with some breeding shortly after the cut, at the expense of structural growth; and others prioritizing growth by delaying breeding for several years post-cut.

Among recaptured wood frogs in the 30m treatment, mean SVL decreased over time, suggesting that frogs invested less energy in structural growth and/or mean age declined over the course of the study. Similarly, females had persistently poor body condition, indicating insufficient food in-take to amass fat reserves. However, buffer treatment did not influence either sex’s mass or male body condition, suggesting recaptured frogs favored maintenance, reproduction, and (among males) fat storage, over growth. Previous research from unbuffered landscapes also found anuran growth constrained in clearcuts versus uncut forest [51, 91, 92]. In the current study, we differentiate between sexes and capture classes, demonstrating that both male and female recaptured frogs experience reduced growth in habitat disturbed by clearcutting, even when frogs can freely move between a 30m buffer, clearcut, and forest beyond the cut. By contrast, new-captured frog size and condition did not differ across treatments, implying that immigrant frogs traversed cuts without significant energetic losses.

In general, spotted salamanders experienced stronger negative effects in the 30m treatment than wood frogs. We suggest three explanations for this inter-species difference. First, both species migrate on rainy nights when desiccation is unlikely [11, 93, 94], but wood frogs are more vagile [88, 95, 96] and may cross cuts more quickly [10, 11], spending fewer days exposed to severe clearcut conditions. Further, salamanders may be more likely to linger in clearcuts, because salamanders primarily shelter in underground burrows [97–99]. In our cuts, stumps were mostly left in place and no mechanical site preparation occurred, such that burrow structure may have been largely preserved [11]. Because aboveground weather is more extreme in clearcuts than forests [10, 65, 67], salamanders in cuts may be trapped in burrows for extended periods, minimizing foraging, and thereby negatively impacting size and condition [70, 100]. Wood frogs, however, frequently shelter in leaf litter [93, 101]. Since young clearcuts have less litter than forests [51, 102, 103], frogs likely minimized time in cuts, only entering to migrate through to distant forests [10]. Finally, spotted salamanders may be more sensitive to terrestrial density dependence than wood frogs. Though both species may crowd into 30m buffers, the consequences may be more negative for salamanders for various reasons. For example, burrows are likely scarcer than leaf litter and salamanders may be forced to share burrows or remain unsheltered. Forced sharing may increase agonistic interactions, causing greater stress [80] and physical trauma [104, 105]. In turn, salamanders may limit foraging to avoid competitive interactions [104, 106] or expend more energy while foraging over a broader area [81]. Small salamanders may also be forced into suboptimal edge or cut habitat by larger competitors [49, 51, 107], negatively reinforcing their stature.

#### Implications

Reduced size and body condition are linked to numerous individual traits that can scale up to detrimentally impact local and regional populations. We categorize individual traits into reproductive, performance, and survival effects. Among the reproductive impacts, small size is associated with decreased clutch mass and volume [39, 108], egg size [39, 82, 108], egg nutrition [39], number of eggs [82, 109, 110], mating success [111–113], and survival during breeding [112]; and increased time to maturity and, for salamanders, inter-breeding interval [81, 82]. Poor body condition can alter mating behavior [114, 115], leading to lower reproductive success [115, 116]. Small size can limit performance through reduced stamina [43, 117, 118], jump distance [44, 119], and migration distance [97, 120], which may inhibit an animal’s ability to escape predators or access good-quality habitat. As for survival, small individuals tend to store fewer lipids [82, 86, 121] and dehydrate faster [42, 122], leading to lower survival, especially under severe weather conditions [86, 123, 124]. Body size also influences population spatial structure: small individuals may be competitively excluded from prime habitats [51, 107] or crowd around water sources [125].

Ultimately, individual effects can alter local and regional population dynamics. Small amphibians of poor body condition are vulnerable to extreme weather and other stressors [42, 126, 127], can depress breeding population size through delayed maturity [128, 129] or skipped breeding events [130–132], and may have low reproductive success [39, 108, 127]. A population of vulnerable individuals is likely less resilient to disturbance and other stressors and may rely excessively on immigration or adult survival to persist [20, 133, 134]. Reduced reproductive success may also translate to fewer or less robust dispersers [135, 136], depressing gene flow and altering regional population dynamics. Where a local population siphons immigrants from the regional disperser pool and produces less viable dispersers, it may act as a regional sink. Though total breeding-adult abundance was not reduced, we did find that both species’ breeding-population structure was altered, with fewer recaptured amphibians and female salamanders present at 30m-buffer pools, confirming that this treatment did indeed serve as sink habitat and that reproductive potential was diminished [35].

### Biomass

#### Mechanisms

Analyzing how adult-amphibian biomass varied across treatments is key to understanding how clearcuts alter ecosystem flows and community interactions. Adult spotted salamanders and wood frogs are important predators of forest-floor invertebrates [137–139] and efficiently convert invertebrate to amphibian biomass [64, 140]. In turn, both species are a vital food source for decomposers and larger predators [71, 137, 140] **DDD**. Adults also provide high-quality food to vernal-pool communities via eggmass deposition or if adults perish while breeding [47, 71, 141]. Consequently, both species are an important conduit for the flow of forest nutrients and energy into vernal pools and link multiple trophic levels in both subsystems [47, 141, 142]. As long-lived, fossorial adults, spotted salamanders also enhance soil fertility and stabilize ecosystem fluxes [140]. Despite these contributions, few studies have examined forestry impacts on amphibian biomass. Available studies show amphibian biomass is generally lower in recent cuts, but none included buffers in the study design [102, 143, 144]; but see [71].

In our experiment, clearcutting was associated with reduced amphibian biomass, but more strongly in the 30m than the 100m buffer treatment. In fact for both species, biomass at 30m-buffer pools declined over time, suggesting deteriorating habitat quality or a lagged response to cutting. For wood frogs, biomass and SVL declined in tandem at 30m-buffer pools, suggesting reduced structural growth as the reason for diminished biomass. For spotted salamanders, biomass fell despite some recovery of individual size and condition and relatively stable breeding abundances [35], suggesting no single driver of salamander biomass loss.

At 100m-buffer pools, adult biomass production was mediated by hydroperiod, such that short-hydroperiod, 100m-buffer pools produced much less biomass than short-hydroperiod reference pools. For spotted salamanders, this pattern mirrored adult abundance [35], not size. For wood frogs, no particular driver was apparent, but only females were affected. It is unsurprising that biomass and hydroperiod were related, since hydroperiod is a determinative force in vernal-pool systems, influencing species distributions [55, 145, 146], community composition [147–149] and larval growth [150–152] and survival [100, 153, 154]. It is well established that spotted salamander and wood frog abundance generally increase with vernal-pool hydroperiod [155–157]. If one considers only short-hydroperiod pools, however, the biomass difference between 100m and reference pools is striking. Apparently, cutting degraded habitat quality in the 100m treatment, but this only occurred, or was only apparent, if the population was also stressed hydrologically.

#### Implications

Adult biomass was reduced at 30m-buffer pools and short-hydroperiod 100m-buffer pools, limiting the amount of high-quality food available to amphibian predators and detrivores in and around these pools [71, 141]. Lower biomass also likely means reduced nutrient and energy subsidies from forests into pools and modified food webs in both subsystems [47, 141]. Salamander biomass declines may additionally serve to destabilize ecosystem processes, since their biomass is a long-term storage location for nutrients and energy [140]. With less salamander biomass, resources may flow more quickly through food webs, resulting in more extreme population fluctuations at other trophic levels [71, 140].

### Conclusions

Traditionally, researchers use species occurrence and abundance to assess disturbance impacts [49, 89, 90]. While valuable, such metrics describe population responses, which may only be discernable after multiple breeding cycles [81, 92, 158]. Individualized metrics, like body size and condition, may be more sensitive since population changes only accrue after enough individuals are affected. Individual metrics can forewarn lagged population responses, reveal sub-lethal effects that undermine population resilience, and illuminate mechanisms driving population responses [81, 89, 90]. Condition, in particular, is often used to index habitat quality since it represents individual fat reserves, which are a function of prey availability and the metabolic demands of a habitat [89, 90, 159]. By contrast, biomass is an infrequently used metric that extends abundance data and connects population changes to ecosystem processes [48, 160].

The reduced body size and condition that we observed indicate clearcutting degraded amphibian habitat quality in the 30m treatment. In response, individuals shifted energy allocation away from structural growth and, in many cases, fat storage. Individual costs of energetic redistribution are substantial, but collective costs may be greater, and potentially include constrained local reproductive output and altered regional population dynamics. Our biomass results also suggest that habitat quality declined at 30m-buffer pools, but further indicate synergistic effects of cutting and hydroperiod in the 100m treatment. More broadly, our biomass results imply that clearcuts altered food-web dynamics and ecosystems fluxes, within and between forests and vernal pools. Forest managers wishing to minimize amphibian size, condition, and biomass impacts should use buffers that are greater than 30 m wide and incorporate hydroperiod into management decisions. Where amphibian conservation is a primary objective and hydroperiod is short (i.e., < 4 months; [55, 155, 156]), buffers wider than 100 m may be necessary. Where amphibians are one of several concerns, buffering pools with hydroperiods longer than four months may provide the greatest conservation-investment return. Note that our results describe amphibian response to a single clearcut configuration (i.e., circular, 100-m wide). Different responses might be observed with alternative clearcut designs, but investigating other designs was beyond the scope of our project. Similarly, we did not examine the potential impacts of predator and competitor community composition, microhabitat, or water chemistry on our response variables. Though we recognize this as a potential study limitation, we do not expect that these factors significantly influenced our cross-treatment analyses for multiple reasons. First, we designed our experiment and statistical analysis to minimize the potential impacts of such within-pool factors. Second, all study pools were fish-free and similar with regard to amphibian community composition and basic water chemistry parameters.

Additional research is needed to understand how individual impacts scale up to influence local and regional population dynamics and ecosystem function. Particular attention should be paid to the potential for differential effects across diverse landscapes. While multiple previous studies from various bioregions similarly demonstrate decreased amphibian body size and condition in response to habitat disturbance (e.g., [91, 159, 161]) and weather stress [127, 162, 163], other studies suggest more complex interactions between individual responses and exposure to stressful conditions (e.g., [164–166]). Likewise, though logging often exerts negative effects on amphibian populations, this may not be universal. For instance, populations of species that evolved in frequently disturbed landscapes may adapt more easily to habitat changes wrought by timber harvests. This appears to be the case, for example, with generalist anuran species in the temperate eucalyptus forests of New South Wales, Australia [17]. Different processes may dampen the negative effects of logging in other landscapes.

Our landscape is largely forested and our cuts regenerated mostly undisturbed. Clearcut structure and micro-climate can change rapidly with regeneration [51, 71, 167]. Cuts that are initially unsuitable for amphibians should regain suitability with time [71, 143, 168]. Though cutting strongly impacted individual amphibians, especially in the 30m treatment, certain metrics, like recaptured salamander mass, started to rebound by the study’s end. In this landscape, there seems to be a vulnerability window of 8 to 14+ years post-clearcut, when adult body size, condition, and biomass are reduced and local populations may be particularly sensitive to additional disturbance or stressors. If regeneration continues undisrupted and habitat quality improves, individual traits likely recover and the vulnerability window closes. Lacking additional stressors, local population persistence and abundance may remain relatively stable and regional population dynamics may be little affected. Recent genetic studies from our landscape support this hypothesis. While clearcutting strongly impacted individual amphibians and increased population vulnerability at many of our focal pools, spotted salamander and wood frog populations across the broader industrial forest demonstrated high genetic connectivity, suggesting regional population resilience [169, 170].

Ultimately, forest managers must consider the cumulative impacts of cutting on a landscape and whether additional stressors are likely to compound the local effects of any single cut. Existing practices, including strategic clearcut rotation on a multi-decadal interval [5, 171], may be sufficient to maintain amphibian connectivity with minimal buffering, given current climatic conditions and forest-product demand. If projections for the northeast are accurate, however, and summers become hotter [172] with more frequent droughts [173], while forest harvests intensify [2], landscape resistance to amphibian movement may increase [11, 174] and regional connectivity be disrupted. In this case, buffers will be a critical tool for maintaining local population resilience in forestry-based landscapes. More broadly, we expect stronger negative impacts to amphibian body size, condition, biomass, and connectivity in suburban and urban landscapes, where a greater proportion of the land area is permanently altered and the inter-pool matrix is less permeable [175–177]. In this context, buffers may be essential to local population resilience, but only when combined with conservation of connective habitat to simultaneously ensure the possibility of rescue and recolonization from the broader meta-population [22, 178] and if the design is tailored to the specific region and landscape [17, 19, 179].

## Supporting Information

S1 Appendix Appendix 1. Variance-covariance structure of size, condition, and biomass regression models.
(DOC)
