# Supplementary material for: Despite Buffers, Experimental Forest Clearcuts Impact Amphibian Body Size and Biomass
Source: PLoS One. 2015 Nov 23;10(11):e0143505. doi: 10.1371/journal.pone.0143505 (PMC4658104; doi:10.1371/journal.pone.0143505)
Supplement: S1 Appendix — Variance-covariance structure of size, condition, and biomass regression models. (DOC) [file pone.0143505.s001.doc]

**Appendix 1.** Variance-covariance structure^a, b^ of size, condition, and biomass regression models.

| **Species & Capture Status** | **Sex** | **Biomass (g)** | **SVL/SUL (mm)^e^** | **Mass (g)** | **Body Condition Index^h^** |
| --- | --- | --- | --- | --- | --- |
| ***Ambystoma maculatum*** | | | | | |
| recapture | **F** | varIdent(1|wetland) | varIdent(1|year) | varFixed(mean.hydro); corARMA(q=1) | varExp(sd.hydro|year); corAR1() |
|  | **M** | varIdent(1\|wetland) | varExp(fitted) | varPower(fitted\|year) | varPower(sd.hydro\|year); corAR1() |
| new-capture | **F** | varExp(fitted|year) | varPower(sd.hydro|trt)^f^ | varExp(fitted); corAR1() | varExp(fitted|year); corARMA(p=1,q=1) |
|  | **M** | varExp(fitted\|year) | varExp(fitted); corAR1() | varIdent(1\|wetland); corAR1() | varPower(fitted\|year); corAR1() |
| ***Lithobates sylvaticus*** | | | | | |
| recapture | **F** | varExp(fitted|trt) ^c^ | NA | varExp(cut.year)^g^ | varIdent(1|wetland); corAR1() |
|  | **M** | varIdent(1\|trt) | varIdent(1\|wetland); corAR1() | varExp(cut.year\|trt); corAR1() | varIdent(1\|year); corARMA(p=2) |
| new-capture | **F** | NA | varPower(sd.hydro|trt); corAR1() | varPower(mean.hydro|trt); corARMA(p=1,q=1) | varPower(sd.hydro|year); corAR1() |
|  | **M** | varFixed(mean.hydro)^d^ | varPower(mean.hydro\|trt); corARMA(p=1,q=1) | varPower(mean.hydro\|trt); corAR1() | varPower(fitted\|wetland); corARMA(p=1,q=1) |

^a^ See Pinheiro and Bates (2000) for descriptions of the possible variance-covariance structures.

^b^ If no structure listed, that component of the variance-covariance structure was not needed.

^c^ Trt = cutting treatment, a categorical variable with three levels: reference, 30m buffer, 100m buffer.

^d^ Mean.hydro = mean pool hydroperiod (days)

^e^ SVL = snout-vent length; SUL = snout-urodyle length.

^f^ Sd.hydro = standard deviation of the mean pool hydroperiod (a measure of pool hydroperiod variability, in days)

^g^ Cut.year = dummy variable representing the difference between the reference treatment and the two cut treatments, over the six study years.

^h^ Index is a measure of relative energy reserves and is calculated as the residuals of an ordinary least squares regression of mass on SVL/SUL. Mass and SVL/SUL were square-root transformed for salamanders and log-transformed for frogs, prior to the regression.
